# Optimizing Digital Health Tools for Colorectal Cancer Screening Uptake in Federally Qualified Healthcare Centers: Insights From the Consolidated Framework for Implementation Research and Technology Acceptance Model

**DOI:** 10.1007/s40615-025-02772-4

**Published:** 2026-01-15

**Authors:** Aldenise P. Ewing, Dede K. Teteh-Brooks, Portia Zaire, Nicolas Nelson, Subhankar Chakraborty, Cathy Meade, Clement K. Gwede

**Affiliations:** 1 The Ohio State University College of Public Health, 1841 Neil Ave, 293 Cunz Hall, Columbus, OH 43210, USA; 2 MD Anderson Cancer Center, Houston, TX, USA; 3 The Ohio State University College of Nursing, Columbus, OH, USA; 4 Southeast Healthcare, Columbus, OH, USA; 5 The Ohio State University Wexner Medical Center, Columbus, OH, USA; 6 Moffitt Cancer Center, Tampa, FL, USA

**Keywords:** Colorectal cancer screening, Electronic health records, Patient portals, Digital health interventions, Federally qualified healthcare centers

## Abstract

**Background:**

Patient portals integrated within electronic health records (EHRs) offer a scalable strategy to promote colorectal cancer (CRC) screening by providing access to education, appointment scheduling, screening reminders, and enhanced communication. However, little is known about how patients and personnel in Federally Qualified Health Centers (FQHCs) engage with these tools to support CRC prevention.

**Methods:**

This convergent mixed-methods study assessed barriers, facilitators, and recommendations related to patient portal use and CRC screening promotion in three FQHCs in the Midwest. Surveys and semi-structured interviews were guided by the Consolidated Framework for Implementation Research (CFIR) and the Technology Acceptance Model (TAM). Eligible participants included patients aged 45–75 and personnel engaged in patient care or portal use.

**Results:**

Seventeen patients and 17 personnel completed surveys and interviews. Most patients (74%) and personnel (71%) found the portal easy to use, and 79% of patients believed it would support cancer screening scheduling. Nearly all personnel (94%) supported portal implementation and were willing to promote CRC screening through it. Identified facilitators included accessible design, embedded education, and alignment with clinical workflows. Barriers included limited access, low digital literacy, and competing staff demands. Participants offered strategies to improve usability, promote culturally relevant content, and deliver preparatory education via the portal. Feedback on a digital CRC screening video suggested strong motivation to screen and support for future integration into portal workflows.

**Conclusion:**

Patient portals are feasible and acceptable tools for increasing CRC screening in FQHCs. Implementation strategies addressing access, literacy, and content relevance are needed to optimize portal-based interventions.

## Introduction

Colorectal cancer (CRC) remains one of the most preventable cancers through screening that leads to early detection and early treatment [1]. Despite recommendations by the United States Preventive Services Task Force (USPSTF) in 2021 to begin CRC screening for all average-risk adults at age 45 [2], studies indicate that screening uptake still lags for some groups, possibly contributing to later-stage diagnoses, increased treatment-related costs, and poorer outcomes [3–5]. Federally Qualified Healthcare Centers (FQHCs), safety net facilities that provide services on a sliding fee scale, report some of the lowest CRC screening rates—currently at just 41% vs. 70% nationally [6, 7]. These centers often serve patient populations facing pronounced structural, social, and economic barriers that further limit access to CRC screening services, underscoring the need for targeted interventions to improve screening-related outcomes.

Technology-based interventions have shown more promise than usual care in promoting CRC screening utilization [8–13]. Usual care typically involves conversations initiated during annual wellness visits or electronic health record (EHR) alert flags only viewable to clinic personnel. The patient portal—a secure online platform embedded within EHRs—provides patients with 24-hour access to their personal health information, including test results, preventive screening reminders, health education resources, appointment scheduling, and direct communication with healthcare providers. This system offers a scalable approach to engage patients in preventive care by facilitating timely information access and ongoing patient-provider interaction [14]. This tool has been associated with improved use of preventive services, including CRC screening, particularly when reminders and tailored educational content are delivered through the portal [15]. However, portal access and engagement are not uniform across all healthcare centers.

The existing literature on patient portal use focuses on commercially insured or health system–affiliated populations [8]. There is limited understanding of how patients receiving care at FQHCs—who often face challenges related to technology access, low digital literacy (i.e., limited skills to navigate online tools) [16], and health system navigation [17]—experience and use patient portals for CRC prevention. Understanding these experiences is particularly important for advancing cancer prevention, as patient portals represent a scalable tool to promote CRC screening, reduce preventable deaths, and improve screening access.

To guide this investigation, we used the Technology Acceptance Model (TAM) to inform the design of the survey questionnaire and applied the Consolidated Framework for Implementation Research (CFIR) to frame the qualitative data analysis. CFIR allows for exploration of multilevel implementation factors, innovation, including inner and outer setting, individual characteristics, and the implementation process—while TAM provides a foundation for understanding users’ perceptions of technology usefulness and ease of use.

This mixed-methods study was designed to explore the experiences, challenges, and recommendations related to CRC screening and patient portal use—both current and future—among patients and clinic personnel across select FQHC sites. By identifying key barriers and facilitators from both patient and clinic personnel perspectives, our findings aim to inform the design of future interventions that will leverage the patient portal to improve screening uptake in real-world primary care settings.

## Methods

Methods are reported in alignment with the *COnsolidated criteria for REporting Qualitative research* (COREQ; see Appendix A) [18].

## Domain 1: Research Team and Reflexivity

### Personal Characteristics

Interviews were conducted by study staff from diverse racial, ethnic and gender-identity backgrounds. Interviewers were the principal investigator and one student, who both taught qualitative research courses. All interviewers underwent mock interview sessions with the semi-structured interview guide to familiarize themselves with the questions and practice probing techniques before engaging with participants in pairs (i.e., one facilitator and one notetaker). Having both a facilitator and a notetaker is a critical part of the qualitative research process. The facilitator helps guide the conversation and ensures the participant’s voice is heard, while the notetaker captures detailed notes, including non-verbal observations, that strengthen the accuracy and depth of the data interpretation [19].

## Relationship With Participants

No prior relationships were established with patient participants before the study commenced. However, existing relationships between the research team and FQHC personnel helped facilitate participant recruitment and collaboration with participating FQHCs. All participants were informed about the research team’s affiliation with the academic institution and the purpose of the study. Interviewers explained that while they may not share the participants’ specific experiences, the study purpose was to understand participants’ insights to improve the current and future use of online patient portals for CRC screening promotion among patients receiving services at FQHCs. This approach helped to build rapport and encourage open discussion during interviews.

## Domain 2: Study Design

### Theoretical Frameworks

Results were interpreted using the TAM and CFIR frameworks to explore relevant constructs related to technology acceptance and implementation of future patient-portal based interventions.

The Technology Acceptance Model originates from the Theory of Planned Behavior and Theory of Reasoned Action and has evolved to become a key model for determining user acceptance of technology [20]. Core constructs of the TAM suggest that **intention** to use technology is influenced by **perceived usefulness** and **perceived ease-of-use** [21]. Extended versions of the TAM suggest that technology use behavior is also mediated by external variables (i.e., individual differences, social influences, system characteristics, and facilitating conditions).

The Consolidated Framework for Implementation Research provides a structured approach for identifying factors— either facilitators or barriers—affecting the success of implementation strategies for technology-based interventions (i.e., patient portal use for CRC screening promotion). The framework encompasses five broad domains: **Inner Setting** (structural and organizational support needed for implementation), **Outer Setting** (external factors such as patient needs and resources), **Innovation** (adaptability and design of the patient portal for CRC screening promotion), and **Individual** (users’ skills, knowledge, and beliefs about the intervention). **Implementation Process** (activities and strategies used to implement the innovation) [22].

The analytic process was guided by the TAM and CFIR frameworks, providing a structured foundation for examining potential factors influencing EHR-linked patient portal use for CRC screening promotion. Through a thematic analysis approach, key themes from qualitative data were identified and categorized according to constructs from these frameworks. The analysis focused specifically on uncovering barriers, facilitators, and actionable recommendations to optimize future use of the patient portal in promoting CRC screening in FQHCs. This approach allowed for a comprehensive understanding of both the challenges and opportunities within the digital health landscape for CRC prevention.

The TAM informed the development of survey measures assessing **perceived ease of use**, **perceived usefulness**, and **intention to use** the patient portal, while CFIR informed qualitative interview domains to explore multilevel barriers and facilitators. Both frameworks guided thematic coding and interpretation, ensuring that results were systematically mapped to constructs for future implementation planning.

### Participant Selection

Two groups were eligible for this study. Patients were eligible if they were aged 45–75 years and were at average risk for CRC. Patients were excluded if they had elevated CRC risk (e.g., family history of CRC or advanced precancerous polyps, inflammatory bowel disease, pelvic radiation history, or known genetic syndromes such as Lynch syndrome), had no interest in digital communication via the patient portal, or were unable to read, speak, or write in English. FQHC personnel were eligible if they self-reported engagement with patients and/or the patient portal in their professional role.

### Sample Size & Justification

The optimal sample size for mixed-methods studies in cancer prevention research depends on various factors, including study aims, methodological approach, and available resources. For qualitative interviews, saturation is often reached at approximately nine interviews and meaning saturation at 16–24 interviews although smaller samples can still yield valid, in-depth insights [23, 24]. The *information power* of the sample, shaped by the specificity of the population, use of theory, quality of dialogue, and analytic strategy, also informs sample adequacy [25].

In this study, a total of 34 participants completed in-depth interviews and surveys. The sample size was determined by the demographic composition and size of the eligible patient pool across three FQHC sites, recruitment feasibility within the project timeline, available study resources, and the exploratory nature of the research. The study was guided by the TAM and CFIR frameworks, with the sample size allowing for thematic saturation within this relatively homogenous group (all patients self-identified as non-Hispanic Black and receiving care in the same metropolitan region). This sample size was deemed sufficient to capture both breadth and depth of perspectives on patient portal use while accommodating pragmatic constraints inherent in the community-based FQHC context (i.e., staff capacity, in-person patient visits, etc.).

To integrate findings across data sources, we applied a convergent mixed-methods design in which quantitative and qualitative results were analyzed separately and then merged for interpretation [26]. Quantitative results provided context for technology acceptance constructs (e.g., ease of use, perceived usefulness), while qualitative interviews offered deeper insight into patients’ and personnel’s perspective on patient portal use. Integration occurred through a weaving narrative strategy within the Results and Discussion sections, with both sets of findings organized and interpreted using the TAM and CFIR frameworks.

### Setting and Recruitment

The recruitment strategy was multi-faceted, leveraging partnerships with select FQHCs and non-profit agencies serving Central Ohio. Participants were recruited from three FQHCs located in Columbus, Ohio: Heart of Ohio Family Health Center, The Ohio State University Total Healthcare Center, and Southeast Healthcare. These partner sites were strategically selected based on their geographic proximity to the King-Lincoln Bronzeville neighborhood [27]—a historically African American community with documented cancer health disparities [28]. Furthermore, African American adults face the higher CRC-specific morbidity and mortality compared to most other racial/ethnic groups [29].

Clinic sites were located within a 5-mile radius of The Ohio State University Comprehensive Cancer Center. This selection approach was intended to engage clinics that served medically underserved populations and were well-positioned to implement digital interventions in real-world settings. Each FQHC offers primary care and preventive services to a high proportion of African American patients and operates within the catchment area of the academic-community partnership supporting this research. At the time of the study, two sites actively used the Epic MyChart patient portal, while the other site was planning for near future implementation of a patient portal linked to the EHR system.

Recruitment efforts within the FQHCs began with a virtual study kickoff meeting and subsequent dissemination of an IRB-approved flyer via email to clinic personnel by the medical director. Additional strategies included in-person outreach and purposive snowball sampling among FQHC patients. Targeted social media ads were also run. Recruitment took place from June 2022 to November 2023.

Eligible participants consented and completed a screening questionnaire of 20 items, followed by a one-on-one interview conducted either in person at the FQHC or via Zoom, which lasted approximately 45 min. Interviews were audio recorded. Participants also completed a supplemental survey questionnaire of 56 items, which was interviewer-assisted for patients and self-administered for personnel. Participants received a $50 gift card to a commercial retailer for their time.

### Interview Guide Domains and Survey Design

Semi-structured interview guides were developed for both FQHC personnel and patient participants to assess barriers, facilitators, and recommendations related to the use of the patient portal and CRC screening promotion via the patient portal. Guide development was informed by CFIR and TAM, which together provided a theoretical foundation to examine contextual, organizational, and individual-level determinants of technology adoption and implementation.

The interview guides were created by the study principal investigator (Ewing) and pilot-tested with research staff to ensure clarity, coherence, and alignment with study objectives. For FQHC personnel, the interview domains included: (1) current use of the patient portal; (2) perceived barriers and facilitators to patient and staff portal use; (3) changes in technology use following the COVID-19 pandemic; (4) existing outreach strategies for CRC screening; (5) perceptions of a Colorectal Cancer Awareness, Research, Education, and Screening (CARES) video that was culturally tailored for African American audiences, low-literacy, and community clinic settings. This video was adopted from an intervention developed and tested in a randomized controlled trial [30]. Lastly, (6) recommendations for integrating the video into clinic workflows and the patient portal system were also included.

The patient interview guide included domains such as: (1) perceptions and usage of the patient portal; (2) ease of use and required support; (3) facilitators and barriers to communication with providers via the portal; (4) preferences for receiving educational content, including CRC prevention materials; and (5) feedback on the CARES video and suggestions for patient portal-based delivery strategies.

Survey questionnaires were developed for both patient and personnel participants. Survey items were derived from TAM and CFIR constructs and assessed attitudes toward patient portal use, including **perceived ease of use**, **perceived usefulness**, and **intention to use**. Personnel surveys evaluated organizational readiness, workflow integration, and willingness to recommend and utilize the portal for CRC screening outreach.

These mixed-methods instruments (See appendices) were designed to elicit comprehensive stakeholder input and inform future strategies for digital intervention delivery and patient engagement in CRC prevention via the patient portal within FQHC settings.

## Domain 3: Analysis and Findings

### Data Analysis

Interviews were transcribed professionally by the Landmark transcription service. Transcripts were not returned to participants for corrections; however, participants were provided with a summary and given the opportunity to add comments or request data removal at the end of each interview (e.g., debriefing).

Thematic analysis was conducted using a combination of inductive and deductive coding approaches in MAXQDA Analytics Pro (24.6.0). Researchers employed an a priori codebook, developed from the interview guide (deductive), alongside open coding to capture initial impressions and emerging patterns within the data. Codes were then organized into broader themes of barriers, facilitators, and recommendations. This blend of inductive and deductive coding provided a comprehensive thematic structure, balancing grounded insights with targeted exploration of specific research interests.

To ensure reliability, two team members independently coded one patient and one personnel interview, achieving a kappa score of 0.64, which indicated substantial agreement [31]. Discrepancies were collaboratively resolved with input from the PI, leading to refinements in the codebook to improve consistency. Data saturation was considered achieved when new interviews contributed minimal additional information to broad themes.

### Ethical Considerations

Informed consent was obtained verbally before each interview. This study was exempt upon review by The Ohio State University IRB, thus ensuring ethical guidelines were followed throughout the research process.

## Results

A total of 34 interviews were conducted, 17 with patients and 17 with personnel of varying roles (e.g., frontline administration staff or licensed healthcare professionals) (Table 1).

### Participant Demographics

#### Patient Participants (*n* = 17)

The average age of participants was approximately 57 years (range: 47 to 67). More than half were male (60%). All patients self-identified as non-Hispanic Black race. Many participants reported annual incomes less than $15,000 (74%). Participants reported varying educational attainment of less than high school (21%), high school graduate (32%), some college (16%), 2-year degree (11%) or 4-year degree (11%). Most (90%) reported Medicaid for healthcare coverage. Participants reported being unemployed and looking for work (42%), disabled or unable to work (26%), full-time (16%), part-time (11%) or being retired (5%). Only 26% of participants were enrolled in the patient portal at time of study participation with those not enrolled primarily stating the reason as ‘*never receiving enrollment information’* (79%). More than half (56%) reported a history of CRC screening and 70% reported screening via FIT. Only half were screened less than a year ago (50%).

#### FQHC Personnel (*n* = 17)

The average age of clinic personnel was approximately 45 years (range: 33 to 59). Most respondents identified as female (77%) and either White (47%) or Black (53%) race/ethnicity. The majority were full-time clinic personnel (94%). Many participants were either licensed healthcare providers (65%) or frontline staff (35%).

### Thematic Analysis

This study explored barriers, facilitators, and recommendations for patient portal use to promote CRC screening among patients at FQHCs. Survey questions provided exploration of TAM-based constructs (Table 2). Qualitative interviews were transcribed, and codes were organized according to CFIR domains: *Innovation*, *Inner Setting*, *Outer Setting*, *Characteristics of Individuals*, *and Process*, with barriers, facilitators, and recommendations highlighted under each section (Table 2).

### Technology Acceptance Model

#### Perceived Ease-of-Use

The majority of patients found the patient portal easy to use, with 58% strongly agreeing and 16% somewhat agreeing. When asked whether it would be easy to learn how to operate the portal, 32% strongly agreed and 37% somewhat agreed. Many clinic personnel also found the patient portal easy to use, with 24% strongly agreeing and 47% somewhat agreeing. Additionally, most indicated they would have no difficulty adapting to using the portal, with 53% strongly agreeing and 41% somewhat agreeing.

#### Perceived Usefulness

A large proportion of patients strongly agreed that the portal would be helpful for scheduling both initial (58%) and repeat (58%) cancer prevention screenings. When clinic personnel were asked whether the portal posed challenges to their work, 12% strongly disagreed, 41% somewhat disagreed, 29% neither agreed nor disagreed, and 18% somewhat agreed. Most clinic personnel believed the patient portal improved clinic care, with 59% strongly agreeing and 29% somewhat agreeing.

#### Intention To Use the Patient Portal

Assuming they had access, most patients indicated they would use the patient portal, with 61% strongly agreeing and 25% somewhat agreeing. Most clinic personnel (94%) strongly agreed with the statement, “I support the implementation of the patient portal at my clinic,” and similarly, 94% strongly agreed that they would inform patients about the portal. Additionally, 82% strongly agreed and 18% somewhat agreed that they would be willing to inform patients about cancer screening through the patient portal.

## Consolidated Framework for Implementation Research

### Inner Setting (Structural and Organizational Support Needed for Implementation)

#### Barriers

Participants noted that FQHCs often lacked the necessary resources and staffing to promote and support patient portal use. Staff members were frequently overburdened and unable to dedicate time to helping patients navigate the portal.

#### Facilitators

Some clinics that integrated active promotion of the patient portal into routine care, such as through aftervisit summaries or posters, reported facilitation of portal uptake. Participants at these sites appreciated having clinic staff proactively introduce and encourage portal use.

#### Recommendations

Clinics should allocate resources for patient portal education and technical support. Providing dedicated staff or incorporating portal promotion into existing workflows could improve the effectiveness of these efforts. Implementing CRC screening reminders and prompts and allowing accessibility in the clinic via tablet stations in waiting rooms, may help boost patient enrollment and engagement in the patient portal.

### Outer Setting (External Factors Such As Patient Needs and Resources)

#### Barriers

Several participants mentioned lack of access to smartphones, internet, and basic digital literacy as significant barriers to patient portal use, particularly among elderly and low-income individuals. In addition, cultural preferences for in-person communication over digital tools limited engagement with the portal.

#### Facilitators

For participants who had access to necessary technology, the patient portal’s ability to offer 24/7 access to health information was viewed as a key facilitator. In some cases, those with previous exposure to technology or family members who could assist found it easier to use the portal.

#### Recommendations

To overcome these barriers, participants recommended providing free or low-cost internet access and devices to patients who may otherwise struggle with connectivity. In addition, offering in-person training sessions at FQHCs could increase patient portal usability and comfort with the platform. Addressing concerns around privacy and data security by providing clear, transparent information about data protection was also suggested.

### Innovation (Adaptability and Design of the Technology)

#### Barriers

Participants highlighted the complexity of the patient portal as a significant barrier, particularly for older adults or individuals with limited technological experience. The portal’s layout and navigation were often viewed as confusing, discouraging engagement.

#### Facilitators

A key facilitator was the relative advantage offered by the portal, which allowed for convenient access to medical records, appointment scheduling, and test results. Participants appreciated the ease with which they could check their health information without needing to call or visit the clinic.

#### Recommendation

Participants suggested that simplifying the portal’s interface and improving its user-friendliness could enhance accessibility. Providing tutorials or walkthroughs within the portal itself was also recommended to help new users become more familiar with its features.

### Individual (Users’ Skills, Knowledge, and Beliefs About the Intervention)

#### Barriers

Low digital literacy and lack of confidence in using technology were frequently cited as barriers to portal use. Many older adults reported feeling intimidated by the platform and were unsure how to navigate it. Mistrust of digital systems and concerns about privacy further deterred some patients from engaging with the portal.

#### Facilitators

Participants who had prior exposure to technology or received support from family members or clinic staff reported higher confidence in using the portal. Positive experiences with other digital services also encouraged some individuals to engage with the portal.

#### Recommendations

To address these barriers, participants recommended offering hands-on training and support to build self-efficacy, especially for older or less tech-savvy users. Integrating peer support or community outreach initiatives, where trained individuals assist others with portal use, could also enhance engagement. Furthermore, providing reassurance about the security of personal health data could help alleviate concerns and build trust in the system.

### Implementation Process

#### Barriers

Participants noted that there was often insufficient promotion of the patient portal during routine healthcare visits. Some patients were not aware of its existence or did not receive guidance on how to use it for CRC screening. Additionally, fragmented communication about the portal’s role in screening caused confusion for some patients.

#### Facilitators

When healthcare providers actively encouraged patient portal use and integrated it into their conversations about CRC screening, patients were more likely to engage. Pre-clinic communication, such as appointment reminders sent through the patient portal, also helped facilitate patient portal use.

#### Recommendations

Participants suggested that clinics more effectively integrate patient portal promotion into pre-clinic communication and routine preventative care workflows, such as during flu shot campaigns or other preventive services. Healthcare providers should be champions for patient portal use. Additionally, using multiple communication channels (e.g., text, email, and portal notifications) for sending reminders about screening could improve engagement and follow-through. Participants recommended that digital education materials, such as brief videos or infographics, be made available through the patient portal prior to appointments. This preparatory education could help patients arrive more informed and ready to engage in shared decision-making about CRC screening options.

### Patient and Personnel Feedback on Digital CRC Educational Video

All patients and personnel provided feedback on a culturally tailored educational video (See Appendix D) designed to promote CRC screening. Codes were generated from patient interviews, with personnel feedback providing complementary perspectives on the video’s content, tone, and patient portal implementation potential.

### Patient Feedback

#### First Impressions

Patients widely described the video as informative and attention-grabbing. Several noted that it effectively conveyed the importance of CRC screening and served as a reminder of how long it had been since their last screening, if ever. Many appreciated the family-centered scenarios and that the message applied to both men and women.

#### Perceptions of Target Audience

Most participants believed the video targeted individuals aged 50–75, with some noting it should be updated to reflect the current USPSTF recommendation to begin screening at age 45. Some interpreted the audience more broadly, emphasizing that “everybody,” including symptomatic individuals, could benefit.

#### Memorable Elements

Frequently cited scenes included mention of symptoms such as blood in the stool, and demonstrated family conversations following abnormal screening results. These vignettes helped underscore the value of early detection and familial influence on healthy decision-making.

#### Positive Reactions

Participants appreciated the video’s clarity, simplicity, and educational value. Many said it increased their awareness and motivation to pursue CRC screening, noting that it made the information feel both accessible and actionable.

#### Suggestions for Improvement

While feedback was generally positive, participants offered recommendations to enhance engagement. These included shortening the video for attention span alignment, incorporating subtitles, using non-clinical settings, and providing information about financial aspects of screening. A few also suggested featuring younger individuals and adding clarity around insurance coverage and screening options.

#### Familiar Faces

Opinions varied: some participants believed the inclusion of familiar clinic staff could enhance trust, while others expressed that negative experiences with their FQHC might make such appearances off-putting. This reflected a broader tension between authenticity and clinical neutrality.

#### Preferred Viewing Locations

Suggested dissemination points included mobile phones, patient portals, waiting rooms, grocery stores, public transit signage, and television Public Service Announcement (PSA) style formats. Participants emphasized convenience and repeated exposure as keys to impact.

#### Integration into the Patient Portal

Patients favored delivering the video via the portal, particularly if paired with an option to immediately schedule a CRC screening appointment. This linkage between education and action was seen as a critical strategy for uptake.

#### Motivational Impact

Most patients found the video highly motivating. Its relatable narrative and accessible messaging increased their readiness to initiate or complete CRC screening.

### Personnel Feedback

#### First Impressions

FQHC personnel offered mixed but generally constructive feedback. Some found the video helpful and informative, while others expressed concern that the length was too long. The video was praised for raising CRC awareness, though several noted it should place more emphasis on the importance of screening for average-risk, asymptomatic individuals. Overall, personnel agreed it was effective in fulfilling the educational intent.

#### Perceptions of Target Audience

Most felt the video was appropriately targeted to a diverse audience of adults aged 50 and above, though some acknowledged the importance of aligning with updated screening guidelines for those aged 45 and older. There was discussion around the portrayal of an African American patient with an abnormal result—some felt it risked reinforcing stereotypes, while others believed it was a strategic and culturally relevant choice to increase resonance with a higher-risk population.

#### Memorable Elements

The most memorable scenes included interactions between family members, particularly between brothers and between a husband and wife discussing the ease of stool-based testing. Personnel also highlighted the statistic that 80% of those diagnosed with CRC have no family history, noting it reinforced screening relevance for all patients.

#### What They Liked

Personnel described the video as accessible, relatable, and diverse. They appreciated the visual aids and clear messaging, which they believed could foster patient understanding and engagement.

#### Suggestions for Improvement

Recommended changes included shortening the video, featuring younger adults, and broadening the depiction of screening methods to include options like Cologuard. Some suggested replacing the gastroenterologist with a primary care provider to increase relatability. Personnel also emphasized the importance of explicitly highlighting screening relevance for high-risk groups and updating the age guidelines to 45+.

#### Familiar Faces

Unlike patients, personnel generally supported featuring familiar and local clinic staff in versions of the video. They believed this could increase patient trust and buy-in, though some noted that customizing videos for each clinic may not be scalable due to cost and resource constraints.

#### Preferred Viewing Locations

Personnel recommended a multi-channel strategy including patient portals, clinic tablets, waiting room TVs, and integration during appointments. They stressed that patients should have several opportunities to view the video—before, during, and after clinical encounters—to reinforce the message.

#### Strategies for Integration

Personnel proposed sending the video as a link via text or email to eligible patients (ages 45–75), embedding it within the patient portal near the time of screening, and encouraging providers or support staff to share the video during appointments. They underscored the need to make the video more engaging, linguistically accessible, and more available across settings.

## Discussion

This study explored the barriers, facilitators, and recommendations for both current and future patient portal use and CRC screening promotion through patient portals at select FQHCs. The findings were organized according to the domains of the TAM and CFIR, providing a comprehensive understanding of how individual, organizational, and external factors influence the uptake of digital CRC screening interventions in FQHC patient populations.

The results indicate that the design and features of the patient portal significantly influence user-engagement and overall effectiveness as a tool for promoting health information, including CRC screening. The patient portal’s ability to provide educational materials, scheduling options, and reminders were highlighted as key strengths by both patients and clinic personnel. These features align with the CFIR domain of **Innovation**, suggesting that when the intervention is user-friendly and integrated with educational content, it facilitates engagement. However, technical issues such as difficulties with navigation and login procedures presented substantial barriers, particularly for patients with limited technological literacy.

In addition to discussing the patient portal’s technical features, participants provided detailed feedback on a culturally tailored digital CRC educational video. Both patients and personnel viewed the video as accessible, motivating and informative despite the need to update the age of screening eligibility to align with 2021 USPSTF guidelines. Patients appreciated its clarity, familial storytelling, and emphasis on early detection, while personnel valued its visual aids and relatability across diverse audiences. Although personnel believed familiar faces would be positively received, some patients reported negative clinic experiences, suggesting that while familiar faces may be one strategy, using celebrities instead of local clinicians might be better received. These responses align with the **Innovation** domain of CFIR, underscoring the importance of adaptable, user-centered content in supporting engagement with digital interventions.

These findings underscore the need to simplify the patient portal interface and integrate supportive features like video tutorials. Providing educational content in advance of appointments—delivered through the patient portal—may further activate patients and foster more productive conversations around CRC screening during clinical encounters. Such modifications could further enhance **Perceived Ease of Use**, a core construct of the *TAM*, by making the patient portal more accessible, particularly for those lacking confidence with technology. By aligning these adjustments with patient needs, the intervention could be optimized for promotion of CRC screening.

The support structures and resources within FQHCs play a critical role in patient engagement with the portal. The integration of portal use into clinic workflows and the availability of staff assistance were identified as effective strategies for encouraging patient portal adoption. These findings align with the CFIR domain of **Inner Setting**, illustrating how organizational support can positively influence the perceived usefulness of the intervention. In 2022, FQHCs used over 15 different EHR systems, with eClinicalWorks and NextGen being the most common. Variability in functionality and interoperability among vendors—driven largely by cost and resource constraints [32]—limits customization of patient portals, directly affecting CRC screening support. Notably, at the time of this study, patients could not directly schedule CRC screening through the portal. Instead, they could message their provider to request testing or a referral or schedule a visit with their provider through the patient portal to further discuss screening. Once a referral (e.g., for colonoscopy) was placed, patients were then able to schedule that procedure via EPIC MyChart. This limitation underscores how differences in patient portal functionality may shape patient experiences and influence the scalability of patient portal-based screening interventions. Future research must address these challenges to assess the patient portal’s effectiveness in improving screening rates and reducing barriers to care.

However, limited staffing and insufficient resources were noted as barriers, particularly for clinics serving high volumes of patients with diverse needs. Without adequate personnel to support onboarding and troubleshooting, patient engagement with the patient portal remained suboptimal. The recommendation to increase staffing support and develop patient portal usability programs for both staff and patients could significantly improve portal engagement, aligning with **TAM’s Perceived Usefulness** construct. Embedding digital navigators within EHR systems to provide dedicated portal support, automating routine functions such as digital reminder delivery, and leveraging partnerships with community-based organizations to extend patient support outside the clinic are promising approaches that should be explored in future research. Such strategies could reduce clinician or staff burden while enhancing patient access and engagement. This dual approach of enhancing the clinic support environment while building user capacity could maximize the impact of future patient portal-based interventions.

CFIR’s **Outer Setting** domain encompasses the external factors influencing patient engagement, such as technology access and cultural considerations. Barriers like limited access to smartphones, internet, and language differences were prominent among underserved populations. These barriers highlight the need for tailored solutions that bridge the digital divide [33] and support diverse patient populations.

Recommendations for implementing multilingual options and culturally relevant content within the patient portal illustrate how social and cultural influences shape perceived usefulness and ease of use, while emphasizing the need to adapt interventions based on community context. Offering digital access points at clinic locations (e.g., free wi-fi, tablets or computers) could mitigate technology access barriers, further enhancing patient engagement and CRC screening uptake. These strategies demonstrate the importance of addressing the social, economic and cultural context to make the patient portal accessible and effective for all populations served by FQHCs.

Internet access and technology use are social determinants of health [33–35]. The **Characteristics of the Individual** domain in CFIR highlights how patient demographics and individual attributes influence engagement with digital health tools. Patients with higher income, younger age, stable housing, and basic technology skills were more likely to report use of the patient portal. Conversely, older adults and those facing economic instability or digital literacy challenges reported more struggles to engage with the patient portal. Thus, technology-based interventions are not a onesize fit all approach to CRC screening promotion. With CRC screening now recommended starting at age 45 for average-risk adults, and the widespread adoption of mobile devices among US adults (91% of US adults own smartphones) [36], technology-based health communication could provide sustained engagement from an earlier age throughout the screening-eligible years.

These findings suggest that interventions must be tailored to meet the needs of individuals who face technological, social and economic challenges. Personalized education programs, such as digital literacy workshops and mobile clinics, could build patient confidence and support widespread patient portal usage. Aligning these strategies with the **TAM**’s external influencing factor of **self-efficacy**, which focuses on confidence in using technology, could lead to improved outcomes by enhancing the ability of these groups to engage with the patient portal independently.

CFIR’s **Implementation Process** domain, which focuses on the implementation strategies that facilitate intervention adoption, revealed that structured onboarding processes and consistent follow-up were effective facilitators of patient portal use for CRC screening promotion. Clinic personnel effort to directly engage patients and encourage patient portal use proved beneficial. These findings suggest that well-designed implementation strategies, such as streamlined onboarding protocols and regular reminders via text messages or emails, are crucial for sustained engagement.

Participants emphasized the value of delivering the digital CRC educational video at multiple touchpoints—within the patient portal, during clinic visits, and via mobile messaging—to ensure exposure and promote screening behavior. Personnel suggested embedding the video within appointment workflows and offering a scheduling link immediately afterward. These strategies reflect actionable **process** adaptations that could enhance patient activation and streamline CRC screening promotion.

These strategies align with TAM’s **Behavioral Intention** construct, which underscores the importance of reinforcing positive attitudes and intentions toward technology use. By embedding these strategies into the process of intervention delivery, FQHCs can enhance patient engagement and promote consistent CRC screening uptake.

### Strengths and Limitations

This study has several limitations. First, it was conducted within a single metropolitan area and included only three FQHC sites, which may limit generalizability to FQHCs operating in different geographic or organizational contexts. Second, the sample size was relatively small (*N* = 34); while this may constrain the statistical power of quantitative analyses and the transferability of findings to other populations, it was sufficient to achieve thematic saturation in the qualitative component given the study’s focus, use of guiding frameworks (CFIR and TAM), and the specificity of the participant population. All patient participants self-identified as non-Hispanic Black and were primarily insured by Medicaid. Nonetheless, this reflects the sociodemographic and socioeconomic composition of patients from the participating FQHCs and the study’s aim to explore barriers and facilitators to patient portal use in a population disproportionately affected by CRC-related outcome disparities and historically underrepresented in CRC intervention studies [37, 38]. As such, findings provide valuable insights relevant to Black patients but may be less transferable to other racial and ethnic groups. They also highlight considerations for the future implementation of digital interventions among patients from lower socioeconomic backgrounds but provide an opportunity to explore the influence of specific health-related social needs on CRC screening behavior. Future research should include more racially, ethnically and socioeconomic diverse samples to assess whether similar or distinct patterns emerge. Third, interviews were conducted in English only, excluding non-English-speaking patients who may face distinct barriers to patient portal use, particularly related to language access and digital literacy. Future studies should incorporate multilingual recruitment and data collection strategies to ensure inclusion of non-English speakers and to explore culturally and linguistically tailored digital health interventions. Additionally, given the cross-sectional nature of the study, we were unable to assess changes in patient portal use or screening behavior over time. Finally, some participants were not enrolled in the patient portal at the time of the interview, which may have limited their ability to fully reflect on its features and functions.

Despite these limitations, this study offers important strengths. The use of a mixed-methods design grounded in two complementary implementation science frameworks (CFIR and TAM) allowed for a comprehensive exploration of multilevel factors that may influence patient portal use and CRC screening promotion via the patient portal. The inclusion of both patient and clinic personnel perspectives enriched our understanding of shared and unique implementation barriers. Our recruitment of FQHCs within proximity to historically underserved, predominantly Black neighborhood and The Ohio State University Comprehensive Cancer Center ensured the relevance of findings to communities disproportionately burdened by CRC disparities. Furthermore, the inclusion of participants who were not enrolled in the patient portal and not up-to-date with CRC screening offered critical insights into the perspectives of individuals who are most at-risk of being overlooked by digital health interventions, thereby enhancing the relevance and inclusivity of our findings. These strengths enhance the credibility and utility of our findings for informing future implementation strategies and tailoring digital health interventions in safety-net settings.

### Implications and Recommendations

Studies have consistently identified racial disparities in patient portal access and use, with Black patients being less likely than White patients to be offered or to use patient portals [39, 40]. Barriers such as concerns over privacy, lack of support, and reduced personalization of care have been well-documented [41]. In this context, provider encouragement plays a critical role in facilitating patient portal access and usage [39]. Moreover, the use of mobile devices has been shown to increase patient portal engagement among Black patients [42]. To improve adoption, FQHCs should focus on raising awareness about patient portal benefits, providing enrollment assistance, and offering low, digitalliteracy materials [43].

This mixed-methods study aligns with national findings that suggest EHR-linked patient portals may help facilitate CRC screening utilization [44], however barriers related to technology access, digital literacy, and cultural factors remain insufficiently addressed. Integrating tailored support strategies—such as enhancing patient portal usability, providing technological access at clinics, and offering culturally relevant content—can optimize engagement and outcomes. Future efforts should also focus on increasing staffing support within FQHCs to facilitate patient onboarding and provide ongoing assistance.

Overall, the integration of CFIR and TAM in this analysis provides a comprehensive assessment of the factors influencing patient portal engagement for future CRC screening promotion. By combining insights from both frameworks, this study identifies actionable strategies to enhance the design, delivery, and support of digital health interventions to ultimately improve CRC screening rates among underserved populations.

## Conclusion

In recent years, EHRs and patient portals have emerged as a promising tool to enhance engagement in preventive care, including CRC screening [8, 10–13]. These digital platforms can offer accessible information, reminders, and scheduling capabilities that can help overcome traditional barriers like transportation, long wait times, and limited clinic availability [45]. Our study extends this literature by highlighting how patients and personnel in FQHC settings—where screening rates remain below the national average—perceive both the promise and persistent challenges of patient portal–based engagement. These insights underscore the need for strategies that not only enhance patient portal functionality but also address digital literacy, technology access, and culturally relevant communication, and point to future research opportunities to design and evaluate multilevel interventions that pair patient portal-based tools with tailored support to overcome digital and structural barriers to CRC screening in underserved populations.

Future efforts should prioritize implementing and evaluating these strategies to close gaps in preventive care access and improve CRC screening rates (41% in FQHCs vs. 71% nationally). Leveraging the patient portal as a platform to deliver preparatory educational materials—such as culturally tailored videos or checklists—may further enhance understanding, encourage screening requests, and promote shared decision-making. By integrating these approaches, patient portals have the potential to become a vital tool for improving CRC outcomes and reducing disparities in preventive care.

## Supplementary Material

Appendices

**Supplementary Information** The online version contains supplementary material available at https://doi.org/10.1007/s40615-025-02772-4.

## Figures and Tables

**Table 1:** Demographic characteristics of participants (patients and healthcare personnel)

Characteristic	Patients* (*n* = 20)	Personnel (*n* = 17)

Age, mean (SD)	56.7 (6.2)	45.1 (8.0)
Gender, n (%)		
Male	12 (60)	4 (23.5)
Female	7 (35)	13 (76.5)
Prefer to self-describe	1 (5)	0 (0)
Race/Ethnicity, n (%)*		
African American/Black	20 (100)	9 (52.9)
American Indian or Alaska Native	0 (0)	1 (5.9)
White	0 (0)	8 (47.1)
Education Level, n (%)		
Less than High School	4 (21.1)	N/A
High School Diploma	6 (31.6)	N/A
Some college or 2-year degree	5 (26.3)	N/A
4-yeear College degree	2 (10.5)	N/A
Other	2 (10.5)	N/A
Missing	1 (5)	N/A
Employment Status, n (%)		
Employed full-time	3 (15.8)	16 (94.1)
Employed part-time	2 (10.5)	0 (0)
Unemployed and looking for work	8 (42.1)	0 (0)
Disabled or Unable to work	5 (26.3)	0 (0)
Retired	1 (5.3)	0 (0)
Other	0 (0)	1 (5.9)
Missing	1 (5.3)	–
Annual Household Income, n (%)		
≤ $15,000	14 (73.7)	N/A
34,999	2 (10.5)	N/A
49,999	3 (15.8)	N/A
Missing	1(5)	N/A
Insurance Status, n (%)		
Other	1 (5.3)	3 (17.6)
Public Insurance (e.g., Medicaid)	17 (89.5)	4 (23.5)
Private Insurance	1 (5.3)	10 (58.8)
Missing	1 (5)	–
Ever Screened for CRC n (%)		
Yes	10 (55.6)	N/A
No	8 (44.4)	N/A
Missing	2	N/A
CRC Screening History n (%)		
Less than a year ago	5 (50)	N/A
Less than 5 years ago	4 (40)	N/A
Less than 10 years ago	1 (10)	N/A
CRC Screening Type n (%)		
FIT	7 (70)	N/A
Colonoscopy	2 (20)	N/A
Other	1 (10)	N/A
Patient Portal		
Patient Portal Enrollment		
Yes	5 (26.3)	N/A
No	14 (73.7)	N/A
Patient Portal is Easy to Use, n (%)		
Strongly Agree	11 (57.9)	4 (23.5)
Somewhat Agree	3 (15.8)	8 (47.1)
Neither agree nor disagree	5 (26.3)	4 (23.5)
Somewhat Disagree	0 (0)	1 (5.9)
Strongly Disagree	0 (0)	0 (0)
Patient Portal would help me schedule cancer prevention screenings (Patients) n (%)		
Strongly Agree	11 (57.9)	N/A
Somewhat Agree	4 (21.1)	N/A
Neither agree nor disagree	1 (5.3)	N/A
Somewhat Disagree	2 (10.5)	N/A
Strongly Disagree	1 (5.3)	N/A
Assuming I have access to the patient portal, I intend to	use it (Patients) n (%)	
Strongly Agree	11 (61.1%)	N/A
Somewhat Agree	5 (27.8%)	N/A
Neither agree nor Disagree	0 (0)	N/A
Somewhat Disagree	1 (5.6%)	N/A
Strongly Disagree	1 (5.6%)	N/A
I am willing to inform patients about cancer screening via the Patient Portal (Personnel) n (%)		
Strongly Agree	N/A	16 (94.1)
Somewhat Agree	N/A	1 (5.9)
Neither agree nor Disagree	N/A	0 (0)
Somewhat Disagree	N/A	0 (0)
Strongly Disagree	N/A	0 (0)

**Note**: Patients were excluded from interviews due to limited English proficiency (*n*=1), duplicate entry (*n*=1), and inability to schedule interview after repeated contact attempts (*n*=1). Participants could self-identify with more than one Race/Ethnicity.

**Table 2:** Barriers, facilitators, and recommendations for CRC screening via patient portal (with example quotes by CFIR domains)

CFIR Domain	Facilitators	Barriers	Recommendations	Example Quotes

Innovation	Accessible technology; integration of educational materials	Navigation difficulties; remembering login credentials	Simplify portal interface; add video tutorials, step-by-step guides	**Facilitator**: PT_18: “That I can see my test results. I can see my lab results. I communicate through messaging and see all my upcoming appointments” **Barrier**: PT_34: “It locks you out quickly if you don’t know your passwords and that stuff.” **Recommendation**: NP_04: “I think having it — excuse me — having an easier way to sign up for the portal. I think it’s difficult having to have a link or a way directly from the doctor’s office. It seems like just the start up is a little bit tricky for people. I know they need to keep it secure, but having an easier way to start process”
Inner Setting	Clinic support; integration of portal into workflows	Insufficient staffing/resources to assist with portal use	Increase staffing; digital literacy programs for patients	**Facilitator**: NP_20: “It’s obvious that it might take a little more time, but it makes patient care more efficient and more quality.” **Barrier**: MD_05: “There really is not a well-thought out plan for support of the patient portal, and so it just becomes another thing that, if it’s being utilized for communication, it’s another thing that clutters the provider inbox, and so providers don’t get a whole lot of support.” **Recommendation**: NP_19: “I think if we had more staffing that would be able to walk people through it more, sit and walk people through it, I think it would be way more into it.”
Outer Setting	N/A	Limited technology access; language differences; cultural barriers	Offer digital access points at clinics; provide multilingual options	**Facilitator**: N/A **Barrier**: NP_02: “We have patients, Somalian patients, some Indian patients. I think it’s mostly in some of the Somalian or Middle Eastern cultures where the men, or the patriarchs of the family, are driving more the conversations and decision-making. In those relationships, if I am caring for the female patient, she may not have MyChart.” **Recommendation**: RN_06: “Another thing maybe could be helpful is maybe a kiosk. I don’t know if a kiosk system would work where—it could be here at Southeast where they can come in and access it for their portal.”
Individuals	Higher income, younger age, tech knowledge	Older age, low digital literacy, socioeconomic barriers	Digital literacy workshops; mobile clinics for underserved groups	**Facilitator**: MD_01: “I would say in my practice, most of the folks are younger. For example, the cystic fibrosis patients I see are very healthcare savvy, very engaged in their care, and they also like—they’re young and they’re tech savvy.” **Barrier**: RN_03: “Definitely the elderly and I guess now that we’re mentioning it, people that are on the lower income scale” **Recommendation**: RN_26: “More tests. Portal. I would really like us to have that portal [crosstalk 08:24] more tests, more just education and basically maybe even a seminar or someone come in, and we get eligible patients to say, “Hey, we have a speaker coming in, and…”
Implementation Process	Structured onboarding; staff encouragement	Inconsistent follow-up	Regular reminders via text/email; streamlined onboarding processes	**Facilitator**: NP_07: “ It might be helpful for them to have a demonstration of this is what the portal is rather than — on a tablet or something. Something that they could maybe even interact with, so that rather than just using the term portal or just talking in generalities, make it very concrete for them, it might help things to click about why it’s important.” **Barrier**: FD_22: “Well, when they come back for their appointment ’cause they don’t really check out. They just walk out the door.” **Recommendation**: PT_09: “It’d be according to how frequently I’m having to go back and forth to the doctors for whatever reason. I wouldn’t want to be inundated with it, but I would like some helpful reminders though, definitely”

## Data Availability

The data generated during this study are not publicly available due to confidentiality and Institutional Review Board restrictions but may be available from the corresponding author upon reasonable request and approval.
