# Supplementary material for: Optimizing Digital Health Tools for Colorectal Cancer Screening Uptake in Federally Qualified Healthcare Centers: Insights From the Consolidated Framework for Implementation Research and Technology Acceptance Model
Source: J Racial Ethn Health Disparities. Author manuscript; Available in PMC 2026 Jun 3. (PMC13231479; doi:10.1007/s40615-025-02772-4)
Supplement: Appendices [file NIHMS2167906-supplement-Appendices.docx]

# Appendix A. COnsolidated criteria for REporting Qualitative research (COREQ).

# Appendix B. Mapping of Interview Guide Topics to Consolidated Framework for Implementation Research (CFIR) Domains

| Instrument | Interview Topic | CFIR Domain |
| --- | --- | --- |
| Personnel Interview | Current use of portal; use of analytics | Inner Setting |
| Personnel Interview | COVID-19 impact on technology; patient use patterns | Outer Setting |
| Personnel Interview | Feedback on CRC video and integration strategies | Innovation |
| Personnel Interview | Workload impact, motivation, empowerment | Characteristics of Individuals |
| Personnel Interview | CRC outreach, resources, messaging platforms | Process |
|  | | |
| Patient Interview | Ease of use, support needs, preferences | Characteristics of Individuals |
| Patient Interview | COVID-19 changes in care delivery and tech use | Outer Setting |
| Patient Interview | CRC screening education and message preferences | Innovation |
| Patient Interview | Preferences for delivery formats and timing | Process |
| Patient Interview | Feedback on video and patient empowerment | Innovation |

# Appendix C. Mapping of Survey Items to CFIR and Technology Acceptance Model (TAM) Constructs

| Instrument | Survey Item Focus | CFIR Domain | TAM Construct |
| --- | --- | --- | --- |
| Personnel Survey | Support for portal implementation | Inner Setting | Behavioral Intention |
| Personnel Survey | Belief portal improves care | Innovation Characteristics | Perceived Usefulness |
| Personnel Survey | Belief patients will support portal | Outer Setting | Perceived Usefulness |
| Personnel Survey | Willingness to inform patients | Process | Behavioral Intention |
| Personnel Survey | Support for self-management and care | Innovation Characteristics | Perceived Usefulness |
| Personnel Survey | Belief portal decreases control | Inner Setting | Ease of Use (inverse) |
| Personnel Survey | Belief portal frees time | Innovation Characteristics | Perceived Usefulness |
| Personnel Survey | Portal is enjoyable/easy/frustrating | Characteristics of Individuals | Ease of Use |
| Personnel Survey | Portal poses work challenges | Inner Setting | Ease of Use (inverse) |
|  | | | |
| Patient Survey | Portal feature usage | Innovation Characteristics | Actual Use |
| Patient Survey | Portal vs. phone communication | Innovation/Inner Setting | Perceived Usefulness |
| Patient Survey | Concern about portal use | Characteristics of Individuals | Ease of Use |
| Patient Survey | Intention to use portal | Characteristics of Individuals | Behavioral Intention |
| Patient Survey | Ease of using/learning portal | Characteristics of Individuals | Ease of Use |
| Patient Survey | Trust in portal security/data | Characteristics of Individuals | Trust |
| Patient Survey | Use for CRC scheduling | Innovation Characteristics | Perceived Usefulness |
| Patient Survey | Portal is a good idea | Characteristics of Individuals | Attitude Toward Use |
| Patient Survey | Barriers to portal enrollment | Outer Setting | External Variables |
| Patient Survey | Use without desktop/laptop | Outer Setting | Facilitating Conditions |
| Patient Survey | Advantages of portal use | Characteristics of Individuals | Attitude Toward Use |

# Appendix D. Digital Educational Video on Colorectal Cancer Screening

As part of the study, participants viewed a culturally tailored digital educational video developed to promote colorectal cancer (CRC) screening among underserved populations. The video was designed to be integrated into patient portals and other digital outreach platforms used by Federally Qualified Health Centers (FQHCs).

**Video Title:** *Colorectal Cancer Screening Saves Lives*
**Video Link:** <https://youtu.be/9pTInOLk9CM>
**Length:** 10:28 minutes
**Production Year:** 2018
**Produced By:** Moffitt Cancer Center
**Purpose:** To increase awareness and motivate average-risk adults to undergo timely CRC screening, highlighting the importance of early detection, common barriers, and screening options.
